# A qualitative study exploring how vocational rehabilitation for people with multiple sclerosis can be integrated within existing healthcare services in the United Kingdom

**DOI:** 10.1186/s12913-024-11424-y

**Published:** 2024-08-27

**Authors:** Blanca De Dios Perez, Vicky Booth, Roshan das Nair, Nikos Evangelou, Juliet Hassard, Helen L. Ford, Ian Newsome, Kate Radford

**Affiliations:** 1 Centre for Rehabilitation and Ageing Research, Queens Medical Centre, University of Nottingham, Room B1387, D Floor, Nottingham, NG7 2RD UK; 2 Mental Health & Clinical Neurosciences, School of Medicine, University of Nottingham, Nottingham, UK; 3 Institute of Mental Health, Nottinghamshire Healthcare Trust, Nottingham, UK; 4 Health Division, SINTEF, Trondheim, Norway; 5 Queen’s University Belfast, Belfast, UK; 6 Leeds Teaching Hospital NHS Trust, Leeds, UK; 7 University of Leeds, Leeds, UK; 8 NIHR Nottingham Biomedical Research Centre, Nottingham, UK; 9 Nottingham University Hospitals NHS Trust, Nottingham, UK; 10 Lay co-author, York, UK

**Keywords:** Vocational rehabilitation, Multiple sclerosis, National health service, Interviews

## Abstract

**Background:**

To explore how a vocational rehabilitation (VR) intervention can be integrated within existing healthcare services for people with multiple sclerosis (MS) in the United Kingdom (UK) National Health Service (NHS).

**Methods:**

Data from 37 semi-structured interviews with 22 people with MS, eight employers, and seven healthcare professionals were analysed using a framework method informed by the Consolidated Framework for Implementation Research and an intervention logic model.

**Results:**

Four themes were identified relating to the structure of current NHS services, how to improve access to and awareness of VR services, the collaboration between internal and external networks, and the benefits of integrating VR within the NHS services. Participants identified several implementation barriers such as poor links with external organisations, staffing issues, and lack of funding. To overcome these barriers, participants suggested enablers such as technology (e.g., apps or online assessments) and collaboration with third-sector organisations to reduce the pressure on the NHS.

**Conclusion:**

Significant organisational changes are required to ensure a successful implementation of a VR intervention within current NHS services. Despite this, the NHS was seen as a trustworthy organisation to offer support that can optimise the health and professional lives of people with MS.

**Supplementary Information:**

The online version contains supplementary material available at 10.1186/s12913-024-11424-y.

## Introduction

Multiple Sclerosis (MS) is a chronic neurological condition characterised by progressive immune-mediated demyelination to the brain and spinal cord [1–3]. Most people are diagnosed with MS during their working lives, typically between 20 and 40 years of age [1]. People living with MS experience a range of cognitive, physical, and psychological difficulties, which can affect their ability to remain at work [4, 5]. The employment rate of people with MS in the United Kingdom (UK) is around 41%, compared to 81% in the general population without disabilities [6]. The reasons why people with MS become unemployed are multifaceted and include both biological (i.e., MS symptoms) and environmental (i.e., attitudes towards disability) factors [7–9].

Vocational rehabilitation (VR) is defined by the British Society of Rehabilitation Medicine (BSRM) as “a process whereby those affected by illness or disability can be enabled to access, maintain or return to employment, or other useful occupation” [10]. Thus, people with MS could benefit from VR to help them manage their symptoms and accommodate their roles and working environment to their needs. However, there is a lack of specialist VR services for people with long-term health conditions in the UK [11, 12].

We developed a job retention VR intervention to support people with MS to remain at work and have tested it in a community setting (i.e., outside of a hospital) [13]. Now we want to explore how this type of VR programme could be integrated into existing healthcare services provided by the UK National Health Service (NHS) because (i) work is good for health [14], (ii) work is an outcome of health interventions of the UK NHS framework [15], and (iii) people are diagnosed with MS in the NHS, which makes it a prime location to identify people in need of employment support soon after diagnosis. Employment for people MS with is associated with improved clinical outcomes such as reduced fatigue and cognitive difficulties, and improved mobility [16].

The UK Medical Research Council (MRC) Framework reports on the need to understand the context where an intervention will be implemented and explore how these interventions will work in practice through logic models (i.e., visual representation of how an intervention works) [17, 18]. Implementation science focuses on understanding factors that impact how a programme or intervention can be integrated into routine practice [19]. Understanding the practical approaches to embed interventions in clinical practice can enhance their efficiency and long-term sustainability and help to bridge the gap between research and clinical practice [20]. Common implementation science strategies involve stakeholder engagement at an early stage to understand the context, offering training and support to facilitate implementation, and advocating for policies that facilitate the long-term sustainability of the programme within the new context [19, 21, 22]. Therefore, it is vital to begin exploring VR implementation issues, given the known time lags to implement complex interventions within healthcare systems [19]. Thus, before we move to the next stage of testing intervention feasibility and effectiveness, this intervention must be adapted to the NHS context.

This study was designed to understand how best to provide VR services for people with MS within existing NHS services. To achieve this aim, we sought information regarding (i) stakeholder’s preferences for VR support and how it could work within the NHS, (ii) “Usual care” for people with MS in the NHS, (iii) barriers and enablers to providing the support within existing NHS services, (iv) long-term impact of integrating VR support within the NHS.

## Methods

### Study design

This was a qualitative study using semi-structured individual interviews following a post-positivist approach to explore the views of key stakeholders on how the NHS could support people living with MS in employment.

### Participants

Three participant groups were recruited for the study (people with MS, employers, and healthcare professionals). Participants were informed that the researcher was interested in exploring how the NHS can offer support with employment to people with MS.

Participants were recruited through convenience sampling using social media (i.e., Twitter), national MS charity groups, and personal contacts. Those interested in the study contacted the primary researcher via email to express their interest. Participants received a £10 voucher as a token of gratitude for their time.

The eligibility criteria for the participants with MS were (i) diagnosis of MS, (ii) working age (as defined by the UK Government), (iii) and currently employed or stopped working in the last 12 months because of MS.

The inclusion criterion for the employers was wide to ensure we recruited a diverse sample. Therefore, the inclusion criterion was currently employing or had experience supporting a person with MS at work. Healthcare professionals were included if they had experience or an interest in supporting people with MS in employment. All participants needed to be able to communicate in English and be willing to consent to participate in the study.

The researcher conducting the interviews (BDP) was a woman from white ethnic background. The researcher works as a Research Fellow, has a background in Psychology (BSc, MPhil, PhD), and has experience working with people with MS, vocational rehabilitation, rehabilitation research, and developing complex interventions. The researcher did not know the participants before their recruitment for the study.

### Data collection

We developed a topic guide for this study for each stakeholder group (people with MS, employers, and healthcare professionals) to support the data collection (supplementary material A). A Patient and Public Involvement (PPI) representative with MS contributed to developing the study documents, interview topic guides, and supported the data analysis process.

All participants were provided with a participant information sheet explaining the purpose of the research and gave written informed consent before data collection. Interviews ranged between 25 and 65 min and were conducted remotely using Microsoft Teams or via telephone. Participants only engaged in one interview, transcripts were not returned to them after the interview, and no non-participants were present. The researcher took notes during the interview on key points raised by the participants.

An intervention logic model [23] was presented to the participants to aid the discussion around what VR is and how it could fit within existing NHS healthcare services.

### Data analysis

Interviews were audio-recorded. Data were transcribed and analysed using a framework method [24, 25] (Tables 1 and 2) informed by the Consolidated Framework for Implementation Research (CFIR) [22] and the intervention logic model. The framework method provides a systematic method to analyse and code data; informing the framework with CIFR facilitates the identification of factors that impact implementation by using the CIFR constructs (i.e., intervention characteristics, inner and outer setting, characteristics of individuals involved, and the implementation process itself) as the coding categories to enhance rigour and transparency of analysis [22, 24, 25]. Including the logic model in the coding framework also facilitated identifying factors that impact implementation specific to our intervention (i.e., Multiple Sclerosis Vocational Rehabilitation “MSVR”) of interest for this study.

**Table 1:** Framework for interviews

Construct	Construct Components
**Consolidated Framework for Implementation Research (CFIR)**	
**Inner setting**: includes features of the implementation organisation that might influence implementation.	**Networks and communications**: The nature and quality of webs of social networks and the nature and quality of formal and informal communications within an organization. **Culture**: Norms, values, and basic assumptions of a given organization. **Compatibility**: The degree of tangible fit between meaning and values attached to the intervention by involved individuals, how those align with individuals’ own norms, values, and perceived risks and needs, and how the intervention fits with existing workflows and systems. **Available resources**: The level of resources dedicated for implementation and ongoing operations, including money, training, education, physical space, and time.
**Outer setting**: includes the features of the external context or environment that might influence implementation.	**Patient needs and resources**: The extent to which patient needs, as well as barriers and facilitators to meet those needs, are accurately known and prioritised by the organisation. **Cosmopolitanism**: The degree to which an organisation is networked with other external organisations.
**Characteristics of individuals**: includes the characteristics of individuals involved in the implementation that might influence implementation.	**Individual stage of change**: Characterization of the phase an individual is in, as they progress toward skilled, enthusiastic, and sustained use of the intervention. **Individual identification with organisation**: A broad construct related to how individuals perceive the organization, and their relationship and degree of commitment with that organization. **Other personal attributes**: A broad construct to include other personal traits such as tolerance of ambiguity, intellectual ability, motivation, values, competence, capacity, and learning style.
**Intervention Logic Model**	
**Activities**: Intervention components and support offered as part of the intervention.	**Vocational Rehabilitation**: Intervention components directly related to vocational rehabilitation and supporting a person to remain, return to or find new employment. **Other intervention components**: Intervention components focused on addressing topics outside of employment (e.g., mental health support, peer support, etc.).
**Outcomes**: Implications of the support offered during the intervention.	**Outcomes for the person with MS**: Direct impact of the intervention on the person with MS. **Outcomes for other stakeholders**: Impact of the intervention on other relevant stakeholders (e.g., careers, employers, healthcare professionals).

**Table 2:** Framework analysis stages and description

Framework Analysis Stages	Description
Familiarisation with the Interview	The Research Fellow (BDP) listened to the audio recordings of the interviews and read the transcripts to familiarise herself with the transcripts. Notes were taken to identify key messages.
Identifying a thematic framework	A thematic framework was developed to organise the data iteratively following the headings of the Consolidated Framework for Implementation Research (CFIR) and the intervention logic model.
Indexing	The interview transcripts were uploaded to NVivo v12 software and the thematic framework was included in the software as nodes to index the data of the interviews. Additional themes not covered by the framework were coded as “other” and revised iteratively throughout the data analysis process to allow for the analysis of ideas not previously identified.
Charting data	A matrix of each theme addressed in the interview was created using NVivo v12 and Microsoft Word to explore the relationship between themes. creating a summary of the information identified and selecting quotes from each theme.
Mapping and interpretation	Through the data charting process, the research team gained an understanding of the data and explored how the data allowed us to answer the research question.

The primary researcher (BDP) coded the interviews and reviewed the findings with the support of the PPI representative, looking for patterns of commonalities and divergence between the different stakeholder groups. The resulting themes were iteratively refined through discussion with the authors. Data that did not fit the framework was coded as “other” to ensure all relevant phenomena identified in the interviews contributed to answering the research question. We used the Consolidated Criteria for Reporting Qualitative Research (COREQ) checklist to improve the reporting of the study (supplementary material B) [26].

## Results

We conducted 37 interviews with 22 participants with MS, eight employers, and seven healthcare professionals. A summary of the characteristics of participants with MS and the employers and healthcare professionals are presented (Tables 3 and 4).

Four themes with ten sub-themes were identified relating to the structure of current NHS services, the characteristics of people with MS accessing the services, and the benefits of integrating VR support within existing NHS services. The interviews were mapped onto CFIR and the intervention logic model via a coding tree (Table 5).

**Table 3:** Demographic, clinical, and employment characteristics of participants with MS

	(*n*=22)
Age [mean (SD)]	44.71 (8.5)
Women	16 (72.7%)
Men	6 (27.3%)
Ethnicity*	
White British	16 (72.7%)
Indian/British Indian	2 (9.1%)
Other white backgrounds	2 (9.1%)
Mixed/multiple ethnic backgrounds	1 (4.5%)
Not provided	1 (4.5%)
**Relationship Status**	
Single	17 (77.3%)
In a relationship	4 (18.2%)
Not provided	1 (4.5%)
**Education**	
A-Levels	3 (13.6%)
Higher National Diploma	1 (4.5%)
GCSE	3 (13.6%)
Degree	9 (40.9%)
Postgraduate	6 (27.3%)
**MS Characteristics**	
Years living with MS	7.0 (6.8)
Years living with symptoms suggestive of MS before the diagnosis	4.9 (4.8)
RRMS	16 (72.7%)
SPMS	2 (9.1%)
PPMS	4 (18.2%)
**Employment characteristics**	
Unemployed	4 (18.2%)
Employed, and working	18 (81.8%)
*full-time*	8 (36.4%)
*part-time*	9 (40.9%)
*On sick leave*	1 (4.5%)
**Job Category**	*n*=18
Level 4 (Professional and managerial)	3 (16.7%)
Level 3 (Associated professional and technical/ skilled trade)	10 (55.5%)
Level 2 (Administrative, caring, leisure, sales, customer service, process, plant and machinery operatives)	5 (27.8%)
Level 1 (Elementary occupation)	0
**Employer Type+**	*n*=18
Private	7 (38.9%)
Public	9 (50%)
Voluntary	2 (11.1%)
Self-employed	2 (11.1%)
**Organisation size**	*n*=18
Large (>250 employees)	11 (61.1%)
Medium (50-249)	4 (22.2%)
Small (10-49)	2 (11.1%)
Micro (<10)	1 (5.5%)
**Employment Sector**	
Healthcare	5 (27.8%)
Financial Services	2 (11.1%)
Transport	2 (11.1%)
Media	2 (11.1%)
Government	2 (11.1%)
Insurance Sector	1 (5.5%)
Education	1 (5.5%)
Tertiary Sector	1 (5.5%)
Engineering	1 (5.5%)
Human Resources	1 (5.5%)

MS: Multiple Sclerosis; RRMS: Relapsing-remitting MS; SPMS: Secondary progressive MS. PPMS: Primary Progressive MS.

Organisation size obtained from UK Government guidelines; Job category obtained from UK Standard Occupational Classification (28); * We use UK Census categories to describe ethnicity

+Out of the 18 participants in paid employment, two were working on two different paid roles

**Table 4:** Demographic and employment characteristics of healthcare professionals and employers

	Employers	Healthcare Professionals
**Gender**		
Women	3 (37.5%)	7 (100%)
Men	5 (62.5%)	0
Ethnicity*		
White British	7 (87.5%)	5 (71.42%)
Indian/British Indian	0	0
Other white backgrounds	1 (12.5%)	1 (14.29%)
Mixed/multiple ethnic backgrounds	0	1 (14.29%)
**Industry**		
Healthcare	2 (25%)	8 (100%)
Financial Services	1 (12.5%)	0
Aerospace	1 (12.5%)	0
Tertiary Sector	4 (50%)	0
**Employer Type**		
Private	2 (25%)	1 (14.29%)
Public	2 (25%)	6 (85.71%)
Voluntary	4 (50%)	0
**Organisation size**		
Large (>250 employees)	5 (62.5%)	6 (85.71%)
Medium (50-249)	0	0
Small (10-49)	3 (37.5%)	1 (14.29%)
Micro (<10)	0	0

Organisation size obtained from UK Government guidelines; Job category obtained from UK Standard Occupational Classification (28); * We use UK Census categories to describe ethnicity

**Table 5:** Coding tree for interviews

Themes and sub-themes	Logic model and CFIR Construct and Components
**Structure of NHS services**	
Service variability	**Inner setting**: *- Available resources* *- Compatibility* *- Networks and communications* *- Available resources*
Readiness for implementation	
The VR Therapist	
Development of internal NHS networks	
Funding for VR Services	
**Improving access to and awareness of VR services**	
Support at the point of diagnosis	**Characteristics of individuals**: *- Individual stage of change* *- Individual identification with organisation* *- Other personal attributes* **Inner setting**: *- Networks and communications*
Raising awareness of VR services	
**Need for the development of external networks**	
Crossing employment and healthcare boundaries	**Outer Setting**: *- Patient needs and resources* *- Cosmopolitanism*
Links with external organisations	
**The benefits of integrating VR support within NHS services**	
Direct implications of VR on the person with MS.	**Intervention logic model**
Benefits for employers and the NHS	

**Fig. 1:**
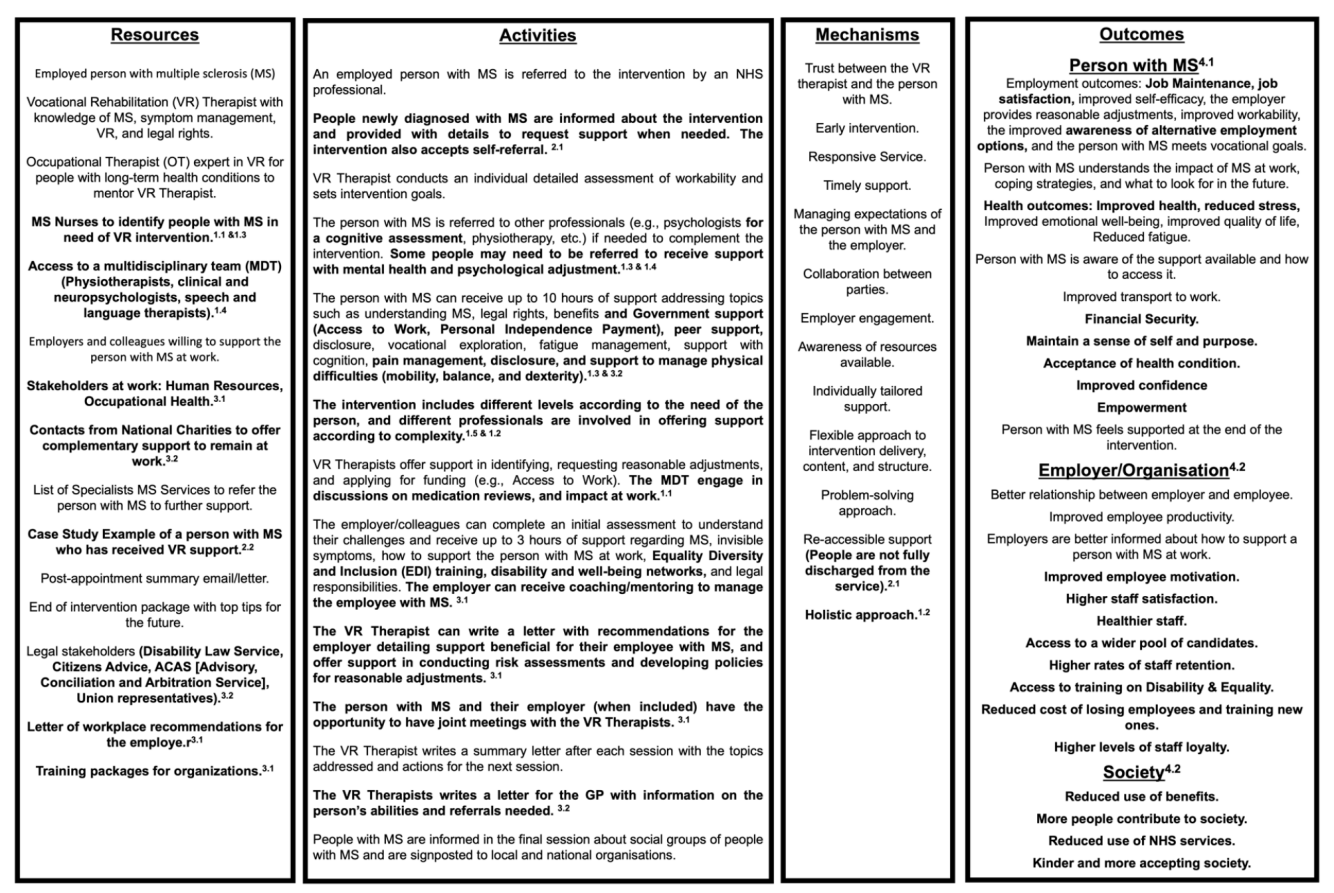
Refined logic model to integrate a VR intervention within the NHS. Intervention logic model representing how a VR intervention for people with MS can be integrated within NHS services. The logic model includes resources needed to deliver the intervention (e.g., person with MS, MS nurse, occupational therapist, links with external organisations, training packages for organizations, etc.), intervention activities (e.g., referral of people newly diagnosed with MS, assessment of employment needs, symptom management), underlying mechanisms (e.g., early intervention, responsive service, holistic approach) and outcomes resulting from the intervention at three different levels: person with MS (e.g., job retention, financial security), employer (e.g., improved workplace relationships, higher staff satisfaction) and society (e.g., reduced use of benefits, reduced use of NHS services)

In a previous study, we developed a logic model following the MRC framework for developing and evaluating complex interventions to understand how the MSVR intervention will work in practice [27]. In this study, participants identified additional intervention components to refine the original logic model (Fig. 1). The statements in bold reflect the changes to the original logic model based on the interviews’ findings. The themes and sub-themes have been labelled with superscripted numbers to link the changes in the logic model with the interviews’ findings (Fig. 1).

### Structure of NHS services^1^

**Fig. 2:**
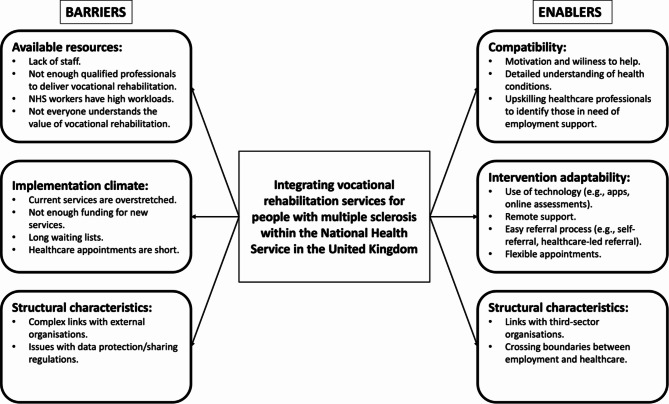
Barriers and enablers to integrating a VR service for people with MS within the NHS in the UK. **Barriers**: Available resources: lack of staff and qualified professionals to deliver the intervention, and no understanding of the value of VR. Implementation climate: Services are overstretched, lack of funding, long waiting lists, and short healthcare appointments. Structural characteristics: Complex links with external organisations, and data protection issues. **Enablers**: Compatibility: Motivation to help, detailed understanding of health conditions, upskilling NHS professionals. Intervention adaptability: Technology, remote support, easy referrals, and flexible appointments. Structural characteristics: Links with third-sector organisations, crossing employment and healthcare boundaries

This theme encompassed participants’ views and knowledge of NHS services available, how a VR service could be embedded within existing NHS services and changes needed in attitudes and the NHS organisational structure for this service to be successful. Participants identified multiple barriers and enablers to implementing the intervention within existing NHS services, such as lack of staff, cost, and time (Fig. 2).

#### Service variability^1.1^

NHS services available for people with MS vary considerably across the UK. Most participants with MS were not aware of what support they could access through the NHS. The participants with MS did not contact their General Practitioner (GP) for MS-related issues, and most of them did not have a designated healthcare professional within their MS-care Neurology team:

I don’t go to my GP for anything to do with my MS…and I have an excellent GP surgery and no problem, but they’re not experts on MS. (MS_08)

Most participants with MS reported having a yearly conversation with their MS Nurse and Neurologist. However, it was common across participants with MS to have no contact for a year with their MS Team, and not all hospitals had access to specialist MS Nurses. The communication with their MS Neurology team increased if their symptoms or circumstances changed:

I’ve been quite fortunate… I’ve had a lot of contact with the MS team, and I think probably because of the number of relapses I’ve had and how severe they’ve been over such a short period of time. (MS_20)

The healthcare professionals discussed the importance of supporting the person remaining at work as part of their usual care. Unfortunately, with services being overstretched and staffing issues, people with MS do not always have access to specialist services:

If we can keep people in work, it’s better, isn’t it? A lot of MS services around the country, I think, only have specialist nurses, and they don’t have the [occupational] therapy support, which is a shame for those clients. (HCP_01)

There were indications that a change of attitude by those who deliver NHS services was also needed for the successful integration of VR services:

I guess it’s just time and its value because I know my MS nurse has got huge amounts of patients on her books and limited time. So, I guess it’s about valuing it [vocational rehabilitation] enough to give it the time. (MS_09)

#### Readiness for implementation^1.2^

Overall, participants saw the NHS as a trustworthy organisation suitable to offer advice on a wide range of topics, including employment:

I think the NHS is a good place because for me it’s trusted information and I think in the modern era, with so much disinformation and misinformation littered all over the place from the Internet. People, just don’t know where to go and a trusted source, so that has to that information has to come from the NHS. (MS_14)

Most participants with MS were not aware of what VR was and the potential benefits that VR could have on their professional lives. They believed that for the integration of the VR service to be successful at the beginning (i.e., receive sufficient referrals), there was a need to raise awareness about the service, for example, through advertisements along with MS-related NHS appointment letters:

The only way you could get information to me reliably would be as some kind of one-page flyer with my invitation to appointment each year. I mean, I know that’s pretty old school, but it’s the only communication I get from my neurology team. (MS_06)

NHS appointments can be short and cover a wide range of medical topics (e.g., medication review, new symptoms). Therefore, the participants explained that the VR service should involve a separate session from the usual healthcare appointment to address employment needs:

If we had this service for example, you go to them as a clinic for your yearly appointment and then tell you, [name], if you would like to have a conversation about your work, you have [VR therapist] in this room or you can arrange a phone call with her for a few weeks’ time whenever it works best for you. (MS_19)

Participants with MS suggested that the VR service could be externally linked to the NHS to improve the likelihood of success:

I think for it to work, there needs to be a designated service and not something that is added to a service that is already there. They’re overstretched already to add another duty. (MS_17)

#### The VR therapist^1.3^

Several participants believed VR support could be delivered by key professionals working within the NHS such as Occupational Therapists (OTs). Participants also suggested support could be provided by MS Nurse Specialists, who were seen as more accessible and knowledgeable about the support available. However, there was an overall view that multiple professionals could be involved in supporting the person with MS at work according to their needs, and there was a drive for involving multiple professionals:

I don’t think a vocational service could run just on occupational therapists, because it’s so individualized and holistic, it has to be an MDT [multidisciplinary team] (HCP_04).

MS Nurse Specialists were seen as essential to identify those who would benefit from VR support from the point of diagnosis. Participants with MS explained that when a person is diagnosed with MS, NHS services should inform the person about the possibility of accessing services to discuss their future with MS at work. MS nurses were seen as essential to initiate this conversation promptly:

I think it kind of needs to be the MS nurse really. Everybody has an MS nurse, and everybody has a vocational OT. Professionals to focus on their vocational skills. Because it puts at the centre that you can still work rather than you’ve got this diagnosis. (MS_07)

OTs could offer specialist support with employment at a later point and should engage with the MS Nurses and other NHS professionals to complement the support. A healthcare professional explained why OTs are essential for this role:

I think occupational therapists should lead [VR] programs just because we look at the individual as a whole. And I think that is super important when it comes to work, with any sort of energy-limiting health condition. It’s not just about what people do at work, it is about what they do outside of work as well, and it’s about getting that balance across work and home life. (HCP_01)

Unfortunately, not all NHS healthcare professionals have the necessary skills to talk about work, or they do not see work as their responsibility, leaving the topic of employment often neglected. This situation leaves people with MS seeking advice with employment from third-sector organisations:

I know that the MS Society have a very useful leaflet for employers, but I don’t know if the NHS do anything similar. (MS_19)

However, some healthcare professionals raised the issue of whether other organisations were suitable for offering this sort of advice:

If you are working independently [from the NHS], I don’t think you would have the skills to complete and deliver research in the same way. And I also think it’s really important for patients because they’re an integral part of the NHS system. (HCP_02)

#### Development of internal NHS networks^1.4^

The VR Therapist delivering the intervention should have good links with the Neurology team responsible for the care of the person with MS. This is particularly important when medications are impacting work performance:

If there are any concerns around medication then we’d be liaising with her [MS Nurse] as well and getting her involved. So yeah, it’s a team approach, but who gets involved at any time depends on where the greater need is I think. (HCP_07)

NHS professionals may lack knowledge about services they can refer people to, and how the referral process works. Having a deeper understanding of other NHS services available and the impact they can have on the lives of their patients can improve the support that people with MS receive:

We get referrals from the acute hospitals and from a smaller rehab hospital. And I think part of it is the fact that there is a rehab medicine rotation. So usually, the doctors have spent some time with us at some point. So, they know what we do. (HCP_06)

Participants believed that the VR service should allow a wide range of referral options (e.g., GP referral, self-referral, etc.) to reduce waiting times and ensure more people access support with employment in a timely manner:

Providing that kind of support [VR] benefits everybody. So, like your primary care, your GP. Making them aware of it so that if they’re coming across people…so I might go to my GP for my MS and my issues that I’m having … And if GPs are aware and on board with something like this, then they’re more likely to kind of refer or get you to self-refer. (MS_04)

#### Funding for VR services^1.5^

Participants saw the VR intervention as a time-consuming, and therefore, high-cost service for the NHS. For this reason, funding was seen as a key barrier to integrating VR services within the NHS:

Having access to this kind of [VR] service means that there’s an added financial burden on the neurology department or other aspects of NHS where they’re already depleting funds. (MS_03)

For the long-term sustainability of the VR service, there is a need to secure funding from key commissioners:

The funding situation at the NHS is extremely tight… There would have to be a strong business case for funding that kind of thing. I do think, because it crosses like health, but also work and you know I think it’s the kind of thing that should be centrally funded. And I think that moves away from the medical model of disability, seeing it as a medical issue would rather than a society disabling people issue. (EMP_03)

To reduce the intervention cost, participants believed that a digital approach (such as having an app with information or remote appointments) could maximise the time that NHS professionals spend engaging in the intervention:

Most people have got some device… You can just do it on [Microsoft] Teams like we’re doing and so I would say though if you do something like a six-week program and then you have an app, so you explain all the resources and then say, I’ll download this, and this will give you the resources that hopefully you might need. (MS_13)

### Improving access to and awareness of VR services^2^

Because most MS participants were unaware of what VR was, they expressed the need to explain to people with MS how VR can help them remain at work. Ideally, this information should be provided at the point of diagnosis, but there were mixed views on this approach.

#### Support at the point of diagnosis^2.1^

The NHS was seen as a crucial organisation to offer VR support because people are diagnosed with MS in the NHS. At the point of diagnosis, people need information about MS and learn about what further support they can access:

It would be worth doing an education session for newly diagnosed so that they know the [vocational] service is there and what type of things you could support them with. There’s a need for early on [support] around awareness and education and knowing that the support is there. (HCP_07)

Some participants believed this information should be provided during the appointment when a person is diagnosed with MS. However, others agreed that most people could feel overwhelmed at diagnosis if informed about the fact that they may struggle to remain at work with MS. Thus, it was suggested that the NHS should provide a second appointment following the diagnosis for those who need it, and leave the option to receive VR support open until the person has come to terms with the implications of the diagnosis of MS:

Just trying to think back to when I was diagnosed. I think when you get diagnosed, you are taking everything in. You’d probably be a bit overwhelmed with everything, so maybe like six months down the line or a year down the line or at least just give out information booklets so that the person can go back to it and read it or…it is difficult because it’s such a sensitive thing. When you get diagnosed and people take it in different ways. Personally, it took me a long time to be concerned with it. (MS_10)

#### Raising awareness about VR services^2.2^

Participants with MS reported challenges accessing NHS services unless their healthcare professionals made a referral or provided them with information about the services. Therefore, significant efforts would need to be made to raise awareness of the service:

It starts with first knowing where to go to get education and information. Then once you’ve got the information, is all about having constructive dialogues and conversations. (MS_02)

Several participants with MS reported not having been actively engaging with the MS-care Neurology NHS appointments because they took an approach to self-manage their condition:

I wanted to try and manage it [my MS] through sort of lifestyle and health. Health changes really if I could. (MS_12)

Therefore, participants reported that some people with MS may reject VR support if their symptoms were considered “manageable” or not too severe:

If you’ve got a diagnosis, but it’s not impacting on your role. In any way you may feel, actually, I don’t really need to disclose because it is, you know, uncomfortable and risky. Even though you’ve got all these protections, it does feel risky to disclose. (MS_07)

NHS professionals need to raise awareness about why learning how to manage MS at work is important to avoid future problems if things change. A healthcare professional explained how their service has multiple referral options to ensure those who need the support can access it:

They can be referred [to our service] by any discipline or any GP consultant, MS Nurse and social workers, basically anybody, and also we do operate a self-referral (HCP_05).

### Need for the development of external networks^3^

For the VR service to be successful, participants reported a need for the NHS to develop networks with external services. These networks could reduce the burden on the NHS and build capacity to provide more comprehensive support.

#### Crossing employment and healthcare boundaries^3.1^

NHS VR services should be able to develop links with the workplace of the person with MS to improve the support offered. Employers may be more likely to provide reasonable adjustments (i.e., modification to the work environment or role to overcome the difficulties experienced by a person with a disability) if information comes from the NHS:

I think we’re quite lucky because we have like our label of the [NHS], and we’re seen as being quite linked up with the MS specialist teams as well. I think we’re quite lucky that when our letters go to occupational health, they’re very like our adjustments are very much like quickly accepted (HCP_03).

The first approach to link with a workplace could be a letter from the NHS reporting the employee’s needs and support that would be beneficial for them:

If an employer could actually say, well, this person’s been diagnosed with XYZ, here’s the report that says, well, this support is needed here. How fantastic would that be? Makes it much easier for the employer to say yes, I can accommodate that. (EMP_05)

Employers also believed the NHS is a prime organisation to advise them on how to manage their employees with health conditions at work, although, they acknowledge the likelihood of this happening was small:

In an ideal world, any information you can receive from the [NHS] professionals is important, but we’re not living in an ideal world at the moment, and I and I know that that is utopia to be able to have the NHS contacting employers for MS… but as I say, in an ideal world, NHS contact would be amazing. (EMP_06)

#### Links with external organisations^3.2^

For the VR service to be successful, there was a need for the service to develop networks outside of the NHS setting. Participants discussed the possibility of involving other organisations or developing a VR service separate from the NHS to overcome the barriers identified:

I think it should be someone who’s a conduit between [work] and the NHS…You know, it’s sort of how you deal with that. And then sort of leave you to work out what you are going to do with it (MS_01).

Most participants mentioned national charities working with people with MS (i.e., MS Society, MS Trust, etc.) as suitable organisations to lead or complement the support provided by the NHS to manage employment needs.

I think charities like the MS Trust and the MS Society are brilliant… They can definitely support in terms of things like publicity with reaching the right people and making sure they’re included in the research. (HCP_02)

Barriers to data sharing between NHS and other organisations may hamper the effective treatment of people with MS:

We all know that the NHS is under pressure. But the centres like ours [MS Therapy Centre] and other facilities across the country can help, and I think it’s just about sharing that information more widely so that other organizations and staff members can access that data and information (EMP_06).

### The benefits of integrating VR support within NHS services^4^

Offering VR support through the NHS was seen as a necessary service that could have a positive impact on the person with MS and their families, employers, society, and the NHS.

#### Direct implications of VR on the person with MS^4.1^

Participants identified multiple benefits for the person with MS and their families resulting from receiving VR support. These have been included in the logic model (Fig. 1).

Participants with MS reported that their work directly impacted their health, but they were not receiving support at work. Participants mentioned that supporting them to remain at work can lead to better clinical outcomes in the future:

I recently found out that I’ve got very high blood pressure…whenever I mentioned that now in my calls with the consultant when they checked up on me, they don’t care…because it’s not clearly MS related or linked and therefore I’m not sure how, how interested they are in widening sort of the net to also include lifestyle and work, even though it’s obviously really important because what if, for example, having a very stressful working life over the next 10 or 20 years causes my MS to progress… (MS_22).

VR support can also have an impact on the well-being and mental health of a person with MS:

I’d say it’s linked to your improved emotional well-being because it’s about self-worth, isn’t it? People that are in employment feel like they have a purpose and the more that can be supported, the longer they will stay in employment if they see the purpose. (MS_09)

#### Benefits for employers and the NHS^4.2^

Participants also identified economic benefits for society. Employers could benefit from VR by improving staff retention rates, and diversity at work:

We found that by talking about our equality, diversity, and inclusion, we have attracted a much broader applicant pool to our jobs, and I think that strengthens our position as an employer. You know, it makes it more likely to find people. (EMP_04)

Those employers who allow their employees to work flexibly (i.e., home working, modifying start and finish working hours), could also see economic benefits in terms of increased employee productivity and lower sick leave rates:

We found as well that people [working from home] were reporting that their need to take sick leave had reduced significantly. Many of our members said they didn’t need as much [sick leave] as they had before because they were able to take their breaks [at home]. (EMP_08)

Finally, supporting people with MS at work can have an economic impact by reducing the number of people on welfare benefits and reduced healthcare use:

The impact of having lots of people on welfare is a huge cost for society. But not only sort of a financial cost, it’s a health cost because again people on benefits have lower health outcomes and that is because they’re financially less secure… having more people with MS and other sort of long-term health conditions in work means that there’s more money in the economy for them to then spend on other businesses and stuff as well. (HCP_01)

The VR service would also mean that the NHS workforce was upskilled to offer advice with employment for people accessing their services.

## Discussion

This qualitative study explored the views of people with MS, employers, and healthcare professionals on how best to integrate VR services for people with MS within NHS services. Although few participants with MS and employers had previous experience receiving support with employment, they believed that offering VR support for people with MS in the NHS could have a positive impact on both employment and healthcare outcomes for people with MS. Healthcare professionals also saw the value of improving healthcare services by providing VR support.

There is evidence that work is good for physical and mental health, with unemployed people experiencing more psychological distress [14]. For people with MS, unemployment is associated with poorer cognitive and functional abilities and greater fatigue [16]. Therefore, a proactive approach to supporting people with MS to remain at work could directly impact health and wellbeing and, by extension, reduce NHS resource use.

The NHS was seen as a trustworthy organisation with trained professionals suitable to offer this support. In particular, the NHS could have a leading role in offering advice on MS and reasonable adjustments to employers. This support is particularly helpful for small to medium enterprises lacking access to occupational health services [28]. Unfortunately, pressure on current NHS services (e.g., lack of staff, long waiting lists) and approaches to healthcare (i.e., medical approach as opposed to biopsychosocial approach) can hamper the integration of VR services within the NHS.

The present study captured several barriers to implementing the intervention within the NHS, such as a lack of staff, insufficient skills to deliver specialist support, poor networks with external organisations, and lack of funding. These are common barriers previously found in the literature exploring how evidence-based interventions can be implemented in healthcare settings [29]. This study also identified potential enablers, such as using technology and upskilling staff to understand the interaction between health and work, to overcome some barriers to successful implementation.

Funding was a barrier to integrating the VR service with the NHS. VR interventions were seen as time-consuming and, therefore, expensive. Future randomised controlled trials exploring the effectiveness of VR should include health economic evaluations that adopt a health and social care perspective, thus providing valuable data to inform commissioning decisions [30]. The cost of VR, in real terms, may be offset by reductions in costs in other parts of the healthcare system (e.g., reductions in GP visits, psychological support services or antidepressant use due to poor mental health because of job loss) or from the wider social perspective (e.g., people in employment paying taxes, rather than dependent on state benefits).

Interventions with a stepped-care approach that offer resource-intense interventions only to those with complex needs could address some of the economic barriers to their integration within the NHS. A wide range of professionals could offer support such as signposting to resources and organisations and VR-trained therapists offering specialist support (e.g., disciplinary meetings, return to work after sick leave, etc.). This finding aligns with recent UK Government efforts to relieve pressure on hard-pressed NHS services. For example, legislative changes enable other healthcare professionals to certify fit notes (i.e., statement of fitness for work), thereby relieving pressure on GPs [31].

This study also explored how to inform people with MS about the availability of VR services at the time of diagnosis. This time was selected because most people with MS are not aware of what VR is and have not received such support [7, 32]. Offering only essential employment information tailored to the needs of the person with MS was seen as necessary so that people with MS realise, early in their journey, that having MS does not mean the end of their professional lives. This finding aligns with research on how to communicate MS diagnosis and how to share information at this critical point without burdening or overwhelming the person with MS [33]. Offering support with employment soon after diagnosis could expedite the return-to-work process after injury and reduce sickness absence and dependency on welfare benefits [10].

Participants suggested that secondary care was the most suitable setting to integrate the VR intervention, reporting that their interaction with primary care was usually limited to issues unrelated to their MS. Even in secondary care, the employment needs of people with MS are typically not identified early, in part because people may not be ready to receive support with employment at the point of diagnosis and because employment status is not routinely recorded as part of their usual care [6]. Additionally, there is a barrier of “expectation” when receiving support in secondary care. Most people attending these services do not expect NHS professionals to address the topic of employment. Services are structured around symptom management instead of adopting a preventative approach to address wider public health and socioeconomic problems such as job retention. Thus, to successfully implement VR within this healthcare setting, there would need to be a biopsychosocial approach to managing people with MS and a shared philosophy that views ‘work’ as a health outcome among the service providers [15].

Issues regarding the organisational structure of the NHS and links with external organisations were clearly illustrated in the interviews. There was a need for the VR service to cross healthcare and employment boundaries. Employers lack awareness about MS and its invisible symptoms and would benefit from advice to manage the needs of the employees with MS at work [7, 32]. Facilitating interaction between employers and VR therapists can help employers understand the implications of a diagnosis of MS at work and provide a platform for addressing their questions. Overcoming the barriers identified in this study requires significant organisational change and is essential to optimise the care that people with MS receive.

Links with other external organisations, such as charities working with people with MS (e.g., MS Society, MS Trust, etc.), can also improve the support people with MS receive. These national charities already offer resources and information to help people live well with MS, and individuals are naturally inclined to seek information on their websites. The role of the third sector in delivering public, social, and health services is growing [34]. Previous research has explored how charities can help deliver complex interventions, such as mental health support to hard-to-reach communities [35], and emotional support to help people with MS at the point of diagnosis [36]. To our knowledge, no research has explored how support with employment could be delivered through the charitable sector in the UK.

One limitation of this study was that most healthcare professionals included were OTs. The study could have benefited from including the views of other professionals, such as MS Nurses, Neurologists, Physiotherapists, and specialist occupational health teams within employer organisations. Another limitation refers to the fact that most MS participants had a degree or post-graduate degree; this limits the generalisability of the findings since people with higher levels of education may have access to better-paid jobs that allow more flexibility than lower paid jobs. Additionally, there is evidence that there are better sickness absence policies for people in better-paid jobs with larger employers [37]. We could have also gained valuable insight by extending involvement beyond those ‘directly involved’ to those ‘passively involved’ [38], for example, by exploring the views of colleagues of employees with MS.

Nevertheless, for this study we used multiple recruitment methods, including social media, national MS charity groups, and personal contacts. This approach allowed us to recruit a wide range of participants, including eight employers. The employers’ views are particularly important given their critical role in supporting people with MS to remain at work. Another strength of this study is the theoretical underpinning of the analysis with the CFIR and intervention logic model, which allowed us to identify implementation barriers at the design stage as suggested by the MRC framework [17, 18]. CFIR has previously been used in research exploring integrating VR interventions within existing healthcare services [39].

## Conclusion

To conclude, this study explored factors affecting the implementation of a VR intervention within the NHS for people with MS. We identified multiple barriers and enablers to implementing VR services within existing healthcare services such as staff shortages, funding for the service, and poor links with internal and external organisations. Changes to the organisational structure of how the NHS works, and moving from a medical to a biopsychosocial approach are needed to support people with MS to remain at work successfully. The findings from this study, including the updated intervention logic model, will be used to refine our original VR intervention and test it within existing healthcare services for people with MS in the UK.

### Electronic supplementary material

Below is the link to the electronic supplementary material.

Supplementary Material 1

Supplementary Material 2

## Data Availability

The datasets used and/or analysed during the current study are available from the corresponding author on reasonable request.

## Abbreviations

CFIR: Consolidated Framework for Implementation Research
EMP: Employer
GP: General Practitioner
HCP: Healthcare Professional
MRC: Medical Research Council
MS: Multiple Sclerosis
MSVR: Multiple Sclerosis Vocational Rehabilitation
NHS: National Health Service
OT: Occupational Therapists
PPI: Patient and Public Involvement
UK: United Kingdom
VR: Vocational Rehabilitation
