# Supplementary material for: A qualitative study exploring how vocational rehabilitation for people with multiple sclerosis can be integrated within existing healthcare services in the United Kingdom
Source: BMC Health Serv Res. 2024 Aug 27;24:995. doi: 10.1186/s12913-024-11424-y (PMC11350982; doi:10.1186/s12913-024-11424-y)
Supplement: Supplementary file 1 — Supplementary Material 1 [file 12913_2024_11424_MOESM1_ESM.docx]

**Supplementary Material A – Interview Topic Guide**

Table S 1 Example of questions from interview topic guides

| Topic Guide | Aim | Example of question |
| --- | --- | --- |
| Participants with MS | - Discuss experiences of MS at work. - Understand usual care and any service gaps. - Explore preferences for VR support. - Identify barriers and enablers to deliver support within the NHS, from their perspective. - Long-term impact of VR support | - What are the current NHS services available for people with MS? - What should the NHS do to help people with MS remain at work? - What outcomes would be particularly relevant to you and others in employment with MS, now and in future? - How do you see support with employment fitting with existing NHS services for people with MS? - What needs to be in place to make this happen?   - When and how should people with MS be referred to this support?   - Who should tell them?/ How should they be informed?   - Who do you think will need these services most?   - Could you think about any situation or characteristics of a person with MS that might make them reject this type of support?   - What characteristics of the healthcare professionals [or other contextual aspects] might help to increase the number of people with MS who agree to receive this type of support? - What are the possible barriers and facilitators to implementing the intervention in the NHS and its sustainability? - What is it about the way the NHS works that can make a difference in how the intervention works? - How would we know if the intervention was working? |
| Employers | - Explore knowledge gaps of employers. - Explore preferences for VR support. - Identify barriers and enablers to deliver support within the NHS | - Can you tell me about your experience supporting employees with multiple sclerosis at work? What support is typically offered to an employee with MS? - What support would help employers improve how they support people with MS to remain at work?   - How should the support be provided? [Prompt: in person, remotely]   - Who should provide it? (OT, nurse, therapist)   - What benefits will these activities provide?   - What problems might be incurred as a result of the activities? - What characteristics of the healthcare system and its professionals [or other contextual aspects] might help to increase the number of employers that agree to receive this type of support? - Who or what is the most appropriate liaison point for initiating/developing support between healthcare professionals and the employer (on both sides)? - Do you foresee any challenges relating to integrating healthcare and employment services? [Prompt: Data protection and communication between healthcare and sectors from the organisation] - Could you think about any situation or characteristics of an employer that might make them reject this type of support? - Do you foresee any challenges relating to the integration of healthcare and employment services? [Prompt: Data protection and communication between healthcare and sectors from the organisation] - What are the possible workplace barriers and facilitators to delivering the intervention for employers and their employees with MS? |
| Healthcare professionals | - Understand the usual care and support available. - Identify barriers and enablers to deliver support within the NHS. - Long-term impact of VR support | - Can you tell me about yourself and your experience with *[MS/VR/employment services, etc]*? - What services are currently available in the NHS for people with MS? - What are the possible barriers and facilitators to the implementation of the intervention in the NHS and its sustainability? - What needs to be in place to integrate the intervention within NHS services?   - When and how should people with MS be referred to this support?   - Who should tell them? / How should they be informed?   - What difficulties might be encountered when incorporating these services within the NHS?   - Who do you think will need these services most? - What outcomes should this intervention achieve?   - How do you think *[insert intervention activity]* can help achieve *[insert outcome]?* - How would we know if the intervention was working? - How should this type of intervention be organised and resourced?   - What would need to happen for MSVR to be incorporated within the NHS?   - How should the intervention be funded? |
